# Mixed Mode Crack Propagation in Polymers Using a Discrete Lattice Method

**DOI:** 10.3390/polym13081290

**Published:** 2021-04-15

**Authors:** Matías Braun, Josué Aranda-Ruiz, José Fernández-Sáez

**Affiliations:** 1 Laboratory of Experimental Mechanics (LABMEX), INTEMA (Research Institute for Material Science and Technology), CONICET, Avda. Colón 10850, 7600 Mar del Plata, Argentina; mbraun@conicet.gov.ar; 2 Department of Continuum Mechanics and Structural Analysis, University Carlos III of Madrid, Avda. de la Universidad 30, Leganés, 28911 Madrid, Spain; ppfer@ing.uc3m.es

**Keywords:** crack propagation, three-point bend, PMMA, lattice model, discrete method, numerical simulation, experimental testing

## Abstract

The fracture behavior of polymeric materials has been widely studied in recent years, both experimentally and numerically. Different numerical approaches have been considered in the study of crack propagation processes, from continuum-based numerical formulations to discrete models, many of the latter being limited in the selection of the Poisson’s coefficient of the considered material. In this work, we present a numerical and experimental analysis of the crack propagation process of polymethylmethacrylate beams with central and eccentric notches subjected to quasi-static three-point bending tests. The developed discrete numerical model consists of a regular triangular lattice model based on axial and normal interaction springs, accounting for nearest-neighbor interactions. The proposed model allows solving the above mentioned limitation in the selection of Poisson’s coefficient, incorporating a fracture criterion defined by a bilinear law with softening that includes the fracture energy in the formulation and allows considering a progressive damage. One of the main objectives of this work is to show the capacity of this lattice to simulate quasi-static fracture problems. The obtained results show that the proposed lattice model is capable of providing results close to the experimental ones in terms of crack pattern, peak load and initial stiffening.

## 1. Introduction

Polymeric materials have been widely used in automotive, aerospace and many other industries during the last decades because of its outstanding mechanical properties, exhibiting a proper compromise between their impact strength and low density and cost. Moreover, thermoplastic polymers have also shown its potential to be used as substitute for metals in a wide range of technical procedures [1,2], and some of them can also be considered as replacements for glass due to their optical properties, such as transparency [3,4]. Within these polymeric materials, polymethyl methacrylate (PMMA) is becoming an increasingly popular material, being used in a very diverse range of fields: electrotechnics, biomedicine, nanotechnology or architecture and furniture [5,6,7,8,9,10].

The fracture behavior of polymeric materials has been extensively studied in last years, in either polycarbonate (PC) [11,12,13], poly-ether-ether-ketone (PEEK) [14,15], PMMA and many others [16,17,18] including new materials obtained by additive manufacturing techniques [19]. Those analysis have been carried out both in dynamic [20,21,22] and quasi-static [23,24,25,26,27] regimes, from an experimental, numerical and analytical point of view, the latter approach being the one in which fractal models are being recently used to investigate the fracture behavior of polymers [28,29,30,31,32]. Many of these works focus on the specific problem of the study of crack propagation direction, pointing out that one of the main disadvantages of PMMA is its susceptibility to break in a brittle or quasi-brittle way, especially when notches, cracks or holes appear [33]. For this reason, the convenience of considering in this work notched specimens with different notch lengths and notch eccentricities is evident.

Due to this growing interest, many studies have addressed the analysis of the crack propagation direction in PMMA, both from an experimental and numerical point of view. Traditionally, numerical models used to study crack propagation have been based on continuum-based numerical formulations, including the Finite Element Method (FEM), or meshless methods [34]. Its major disadvantage is the high computational cost involved in reproducing crack propagation, using techniques such as element removal, re-meshing, cohesive elements or the Extended Finite Element Method (XFEM) [35].

With the clear objective of reducing the aforementioned high computational cost of finite element models, discrete models have been presented as an alternative in which it is not necessary to define the basis function (also called shape functions) on reference elements. Discrete models are those formed by a series of individual points linked through linear elements, so that the total system is shaped like a mesh or grid. The way in which the interaction elements are defined is what distinguishes each discrete model. Included in this category, it is important to mention: molecular dynamics models [36,37,38,39], focusing on the simulation of the movement of atoms and molecules through the definition of atomic potentials; peridynamic models [21,40], a nonlocal form of continuum mechanics in which the equation of motion is replaced by an integro-differential equation where spatial derivatives are removed, the peridynamic bonds transfer forces between connected points and their failure is used to establish damage at a certain point; and finally, the Lattice Models (LMs) [41,42,43,44], which are composed of one-dimensional mechanical elements that connect a set of nodes that may be regularly or irregularly distributed, being their main advantage the simplicity in the description of the propagation of cracks by eliminating the mechanical interaction between nodes.

The above-mentioned lattice models present restrictions on the selection of the Poisson ratio, being this one of the main obstacles to modeling and analyzing a wide range of materials with this type of models. In some LM, the restrictions are related to problems of instability caused by obtaining negative stiffness for certain values of the Poisson coefficient [41,45], while in other models Poisson’s coefficient must be exactly equal to 0.25, in order to ensure consistent equivalence between the discreet and the continuous isotropic [42].

In this work, a 2D lattice model based on Born elastic potential is presented and validated with experimental results carried out on PMMA pre-notched specimens under quasi-static conditions. This model allows to solve the mentioned limitation in the selection of the Poisson’s coefficient, incorporating also a fracture criterion defined by a bilinear model with softening that includes the fracture energy in the formulation and allows to consider a progressive damage. It is important to highlight that vast majority of discrete models present in scientific literature employ fracture criteria based in models without considering the progressive damage of the material, due to the extra difficulty that represents relating the model parameters to the fracture energy. The authors have already proved in previous works [46,47,48] the viability of the presented model to study dynamic crack propagation and branching problems.

This paper is organized as follows: In Section 2, the experimental set-up is shown defining the mechanical properties of PMMA, the geometry of the specimens and the tests methodology. Then, the formulation of the lattice model is presented, taking into account the general equations and the constitutive model, and describing its implementation in order to reproduce the experimental results. In Section 3, the comparison between experimental and numerical results is exhibited, demonstrating the predictive capacity of the proposed discrete model and its accuracy.

## 2. Materials and Methods

### 2.1. Experimental Procedure

**Figure 1:**
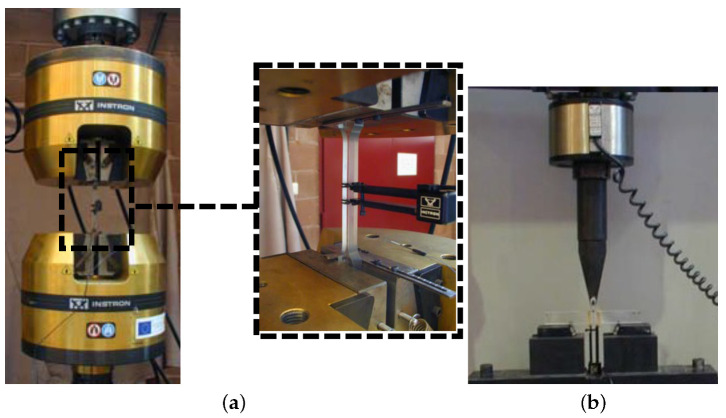
Experimental set-up for (**a**) uniaxial tension test and (**b**) fracture toughness test.

In this work, both characterization and fracture tests have been carried out, in order to analyze the fracture behaviour of PMMA. The mechanical properties, used as inputs in the numerical implementation, are obtained from uni-axial tension and fracture toughness tests following the standard ASTM D 50545 [49]. The developed experimental set-ups are shown in Figure 1, for both conducted tests, carried out in an computer-controlled INSTRON 8516 universal testing machine and using a 100 kN load cell.

**Figure 2:**
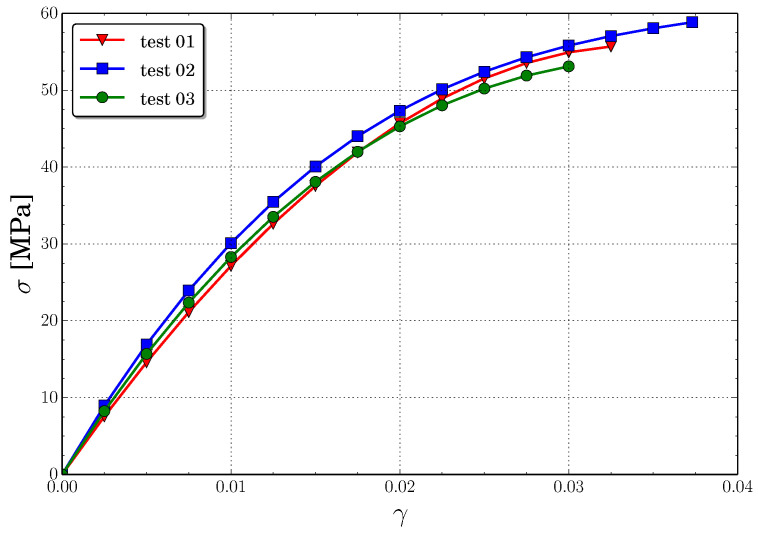
Stress-strain curves obtained from uniaxial tension load.

The true stress-true strain curves obtained from the uniaxial tension test are represented in Figure 2, from where the values of Young’s Modulus and tensile strength are obtained. These mechanical properties are in agreement with the experimental results reported by other authors [23,50,51,52].

The fracture energy $G_{f}$ is calculated as a function of the critical strees intensity factor $K_{IC}$, the Young’s modulus *E* and the Poisson ratio ν:

(1) $$G_{f}=\frac{K_{IC}}{E(1-\nu^{2})}.$$

$K_{IC}$ is defined from the peak load $P_{Q}$ obtained form the fracture toughness experimental test. Table 1 shows the values of the peak load obtained for each specimen, while Table 2 presents the global results from uniaxial and fracture tests and some properties obtained from the data presented in [23].

Once the characterization of the material has been performed, three-point-bending tests have also been carried out, using PMMA notched beams submitted to quasi-static loading conditions. As in the case of those characterization tests, these three-point-bending tests were performed on a computer-controlled INSTRON 8516 universal testing machine under displacement-control mode at normal conditions of pressure and temperature.

**Figure 3:**
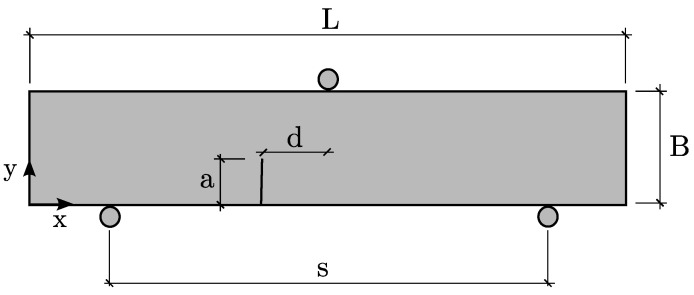
Geometry and boundary conditions of the experimental set-up.

Figure 3 shows a schematic representation of the geometry and boundary conditions of the experimental tests. The beam dimensions were 100 mm in width (L), 20 mm in high (B), and a thickness of 10 mm, while the distance between supports (s) was 80 mm.

Three different initial notch lengths (a=6, 8, and 10 mm) and four notch eccentricities (d=0, 10, 20, and 30 mm) were considered. In order to obtain statistical results, three specimen for each configuration were tested.

**Figure 4:**
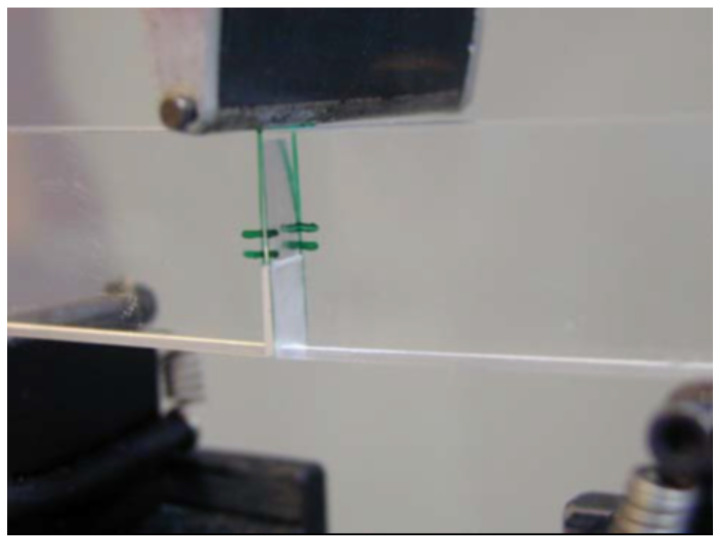
Three point bending test during crack propagation process.

The beams were obtained from a plate of PMMA cut with laser technique, and notched using a diamond sawing wire, creating a 0.28 mm notch-tip radius. Figure 4 shows a centered notch specimen during an ongoing three point bending test.

The results of these three-point bending tests will be shown in Section 3, together with the results obtained from the numerical model proposed in the present work.

### 2.2. Description of the Lattice Model

The motion equation used in this work is developed in [46] and implemented in other works [47,48]. For the sake of completeness, we briefly outline here the general framework and the final formulation of involved equations.

The equation of elasticity which govern the displacement field u in a linear elastic homogeneous material [53] is given by

(2) $$\ddot{u}=c_{t}^{2}\nabla^{2}u+\left(c_{l}^{2}-c_{t}^{2}\right)\mathrm{grad}\;\mathrm{div}\;u,$$

where the transverse $c_{t}$ and longitudinal $c_{l}$ speeds of sound are material properties related to mass density ρ, Young’s modulus *E*, and Poisson’s ratio ν. In the plane strain case, as considered in this work, the expressions for the transverse and longitudinal wave speeds are

(3) $$c_{t}=\sqrt{\frac{E}{2\rho (1+\nu )}},\;c_{l}=\sqrt{\frac{E(1-\nu )}{\rho (1+\nu )(1-2\nu )}}.$$

The model proposed in [46] is based on a decomposition of the displacement field in normal ($u^{n}$) and transversal ($u^{t}$) components. Hence, the differential equation of motion is obtained as

(4) $$\ddot{u}=c_{l}^{2}\nabla^{2}u^{n}+c_{t}^{2}\nabla^{2}u^{t}.$$

**Figure 5:**
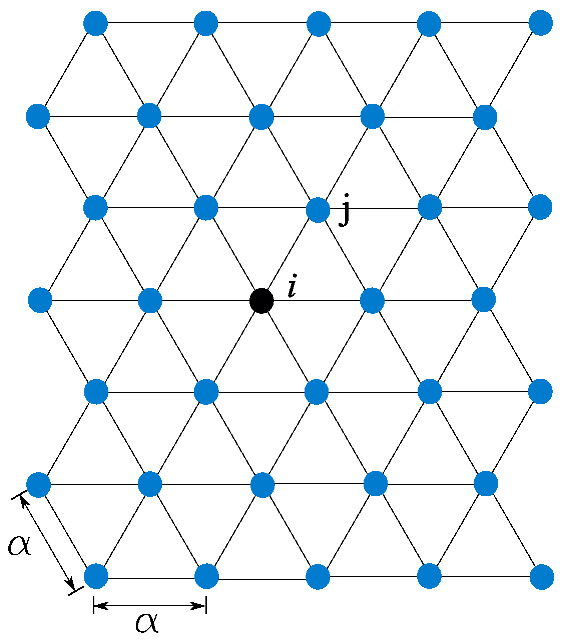
Regular triangular lattice of spacing α.

On the other hand, the discretization method fully developed in [46] yields to Equation (5), for the case of plane strain conditions and a regular triangular lattice of spacing α (see Figure 5),

(5) $${\ddot{u}}_{i}=\frac{2c_{l}^{2}}{3\alpha^{2}}\sum_{j=1}^{6}u_{ij}^{n}+\frac{2c_{t}^{2}}{3\alpha^{2}}\sum_{j=1}^{6}u_{ij}^{t},$$

where $u_{ij}^{n}=\left(u_{ij}\cdot n_{ij}\right)n_{ij}$ is the vector of normal displacement, and $u_{ij}^{t}$ is the vector of transversal displacement, which can be obtained as $u_{ij}^{t}=u_{ij}-u_{ij}^{n}$, being $n_{ij}$ the initial normal unitary vector pointing from particle *i* to particle *j*. Note that Equation (5) is stable for the entire range of values of Poisson’s ratio.

The equations of the model are assembled by enforcing the second Newton’s law at every node. This procedure results in the subsequent system of equations

(6) $$M\ddot{u}+F\left(t\right)-P\left(t\right)=0,$$

where u represents the vector of generalized nodal displacements, **M** the mass matrix, F(t) the vector of internal nodal forces and P(t) the vector of the external nodal loads. The Equation (6) is integrated in the time domain with the Verlet algorithm [54]. To ensure numerical errors do not increase dramatically, time increment is defined according to Courant–Friedrichs–Lewy criterion [55].

**Figure 6:**
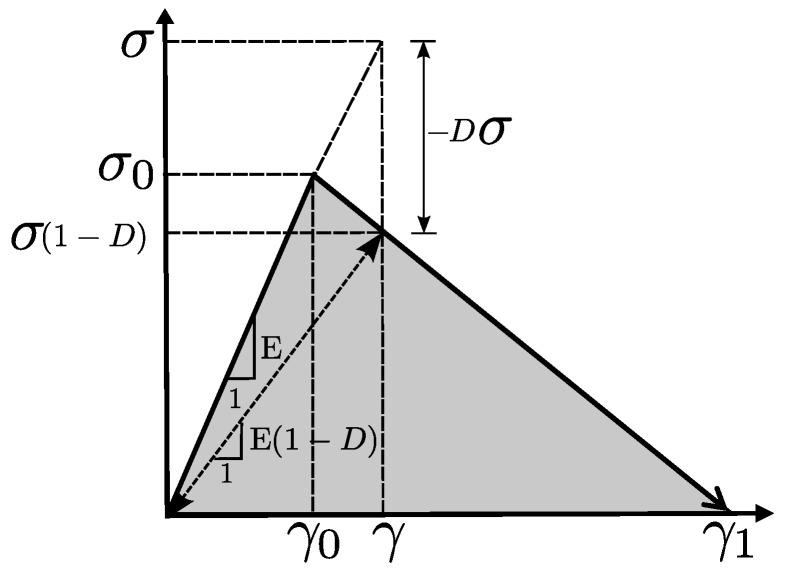
Schematic representation of the bilinear constitutive law adopted to each interaction ij.

Figure 6 shows the stress-strain curve which defines the constitutive model implemented in this work, following the same procedure proposed in [47], where the strain tensor $\gamma_{kl}$ to each node *i* is calculated as [41,46,47,48,56,57,58]

(7) $${\left(\gamma_{kl}\right)}_{i}=\frac{1}{6\alpha }\sum_{j=1}^{6}\left(u_{j}-u_{i}\right)n_{ij}+n_{ij}\left(u_{j}-u_{i}\right).$$

Thus, the strain tensor which defines each interaction ij is approximated as the mean value between *i* and *j* node tensors.

The damage variable *D*, for a bilineal problem with linear softening law, is given by [59]

(8) $$D\left(\bar{\gamma }\right)=1-\frac{\gamma_{0}}{\gamma_{1}-\gamma_{0}}\left(\frac{\gamma_{1}}{\bar{\gamma }-1}\right),$$

where $\bar{\gamma }$ is the effective strain, while $\gamma_{0}$ and $\gamma_{1}$ are parameters which define the strain at the peak stress and at the complete softening stage respectively. The damage variable *D* can assume values between 0 to 1 (D=0 represents the state of non-damage and D=1 represents the onset of fracture). The effective strain is defined as

(9) $$\bar{\gamma }=\sqrt{{\langle \gamma^{p1}\rangle }^{2}+{\langle \gamma^{p2}\rangle }^{2}},$$

where $\gamma^{p1}$ and $\gamma^{p2}$ are the principal strains and 〈·〉 is the Macaulay bracket.

**Figure 7:**
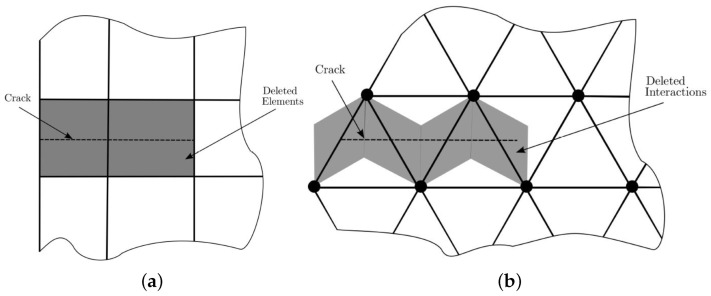
Representation of a crack by deleting (**a**) elements in finite element method and (**b**) interactions in discrete model.

In order to define the constitutive model, we applied the same methodology that the classical formulation of the Element Deletion Method (EDM) implemented in FEM [59]. Figure 7 represents the way a crack was treated with FEM and LM. In the first method, the crack advanced through elements, while in LM the crack crossed interaction bonds. Note that when a bond was broken the equivalent portion of real cracked material corresponds to the area of influence of the interaction.

In discrete models as well as in EDM, it was necessary to define a relation between the surface energy of a crack passing through parallel elements, and the energy associated to the constitutive model (Figure 6). This energy consistency defined the objectivity of the constitutive model with respect to the mesh size.

Hence, we needed to equalize the energy dissipated due to the failure of a real portion of material, with the energy associated to the stress–strain law (shown in Figure 6):

(10) $$G_{f}h_{ij}=g_{f}\Omega_{ij},$$

where $G_{f}$ is the fracture energy, $h_{ij}$ is a characteristic dimension, and $g_{f}$ is the specific energy (energy per unit volume) dissipated during the deformation process:

(11) $$g_{f}=\int_{0}^{\infty }\sigma d\gamma .$$

In a uni-axial deformation process $g_{f}$ would be, for a given point, the area under the stress–strain curve at that point. We can rewrite Equation (10) as:

(12) $$G_{f}h_{ij}=\frac{1}{2}E\gamma_{0}\gamma_{1}\Omega_{ij}.$$

**Figure 8:**
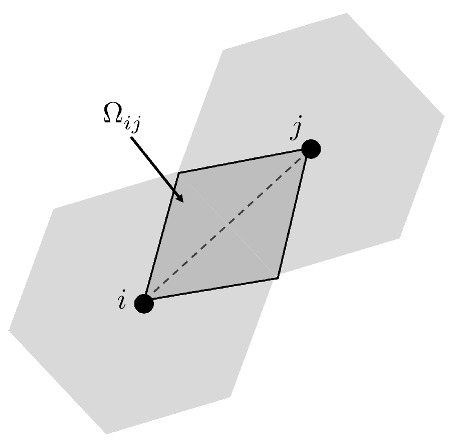
Influence area of each interaction.

Therefore, we can calculate the critical strain $\gamma_{1}$ as:

(13) $$\gamma_{1}=\frac{2G_{f}}{E\gamma_{0}}\frac{h_{ij}}{\Omega_{ij}},$$

where $G_{f}$ is the fracture energy and $\Omega_{ij}$ is the area of influence of the interaction ij (see Figure 8), given by

(14) $$\Omega_{ij}=\frac{\alpha^{2}}{2\sqrt{3}}.$$

In EDM implemented in FEM no information about the orientation of the crack surface generally is included [60]. Instead, the use of square or nearly square elements is common, where the characteristic dimension is adopted equal to the length of the side of the elements [59]. For simplicity in this work we adopt the parameter $h_{ij}$ to be the minimum dimension of the area of influence to the interaction, as

(15) $$h_{ij}=\frac{\alpha }{2\sqrt{3}}.$$

From Equation (13) it is possible to define a coefficient *K* as the relation between $\gamma_{1}$ and $\gamma_{0}$

(16) $$K=\frac{\gamma_{1}}{\gamma_{0}}=\frac{2G_{f}}{E\gamma_{0}^{2}}\frac{h_{ij}}{\Omega_{ij}},$$

where it is necessary to verify that K≥1 to ensure the consistency of the model ($\gamma_{1}\geq \gamma_{0}$). In this way, we can calculate the characteristic length, $\alpha_{cr}$, which preserves the stability of the constitutive model as

(17) $$\alpha_{cr}=\frac{2G_{f}}{E\gamma_{0}^{2}}.$$

In this way, we considered the objectivity by modifying the constitutive law as a function of cell size.

The numerical model was developed through Matlab software, defining all nodes positions and connectivities, reproducing the specimen geometry shown in Figure 3.

**Figure 9:**
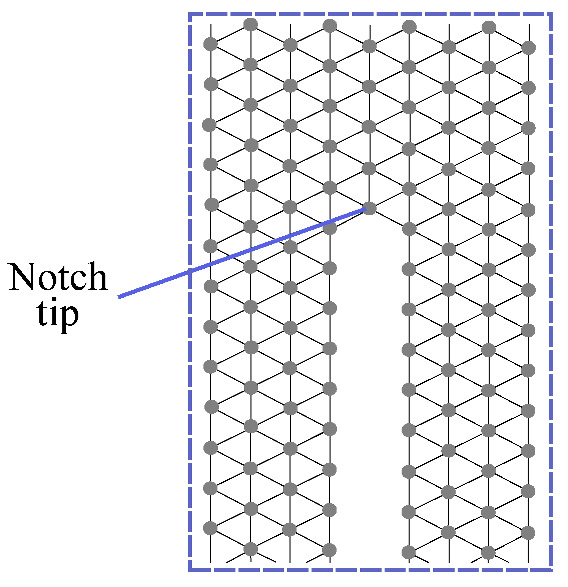
Representation of the lattice discretization near of the beam notch.

Figure 9 presents a more detailed view of the lattice model around the notch area, where it can be seen that the notch is modelled by deleting the nodes localized into it. The beam is discretized using a cell size of α=0.25 mm which is defined after a mesh sensitive analysis, being lower than the characteristic length $\alpha_{cr}$ given by Equation (17).

The external load applied on the beam was modelled as an imposed constant velocity of v=1 mm/s at the central nodes of the upper face, while the vertical displacement of the nodes located on the supports was restricted.

In order to guarantee the quasi-static experimental load condition, the adopted velocity *v* verify that the maximum kinetic energy is lower than the 5% of the total strain energy, for all time increments.

## 3. Results and Discussion

To accomplish the validation of the proposed lattice model for fracture behavior of PMMA, the experimental measurement of applied force versus displacement was compared with the reaction force predicted by the numerical simulations. The initial stiffness, peak loads and crack propagation paths were analyzed and consequent relative errors between experimental and numerical results were calculated.

**Figure 10:**
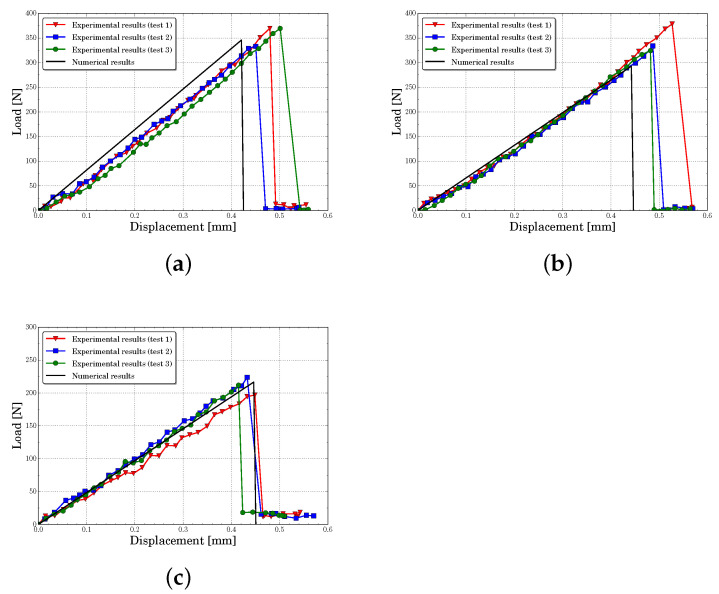
Load-displacement curves for centered notch specimens predicted for different notch lengths *a* (**a**) a=6 mm, (**b**) a=8 mm, (**c**) a=10 mm.

**Figure 11:**
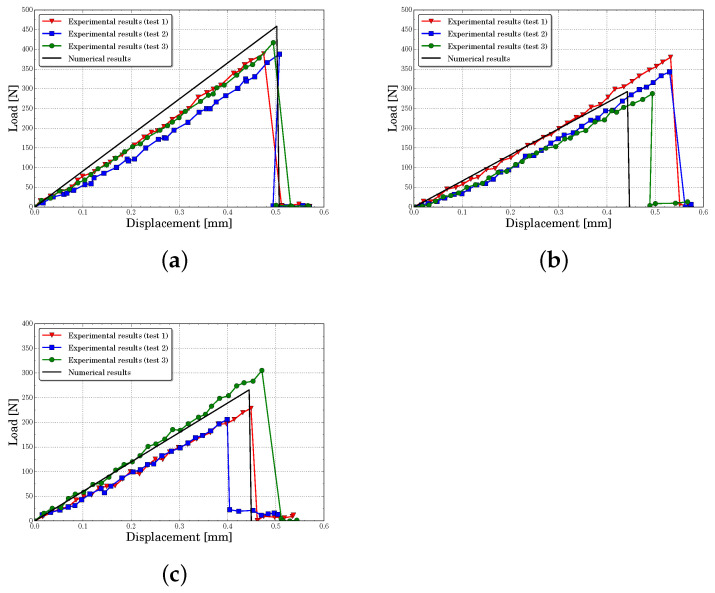
Load-displacement curves for a notch eccentricity of d=10 mm predicted for different notch lengths *a* (**a**) a=6 mm, (**b**) a=8 mm, (**c**) a=10 mm.

**Figure 12:**
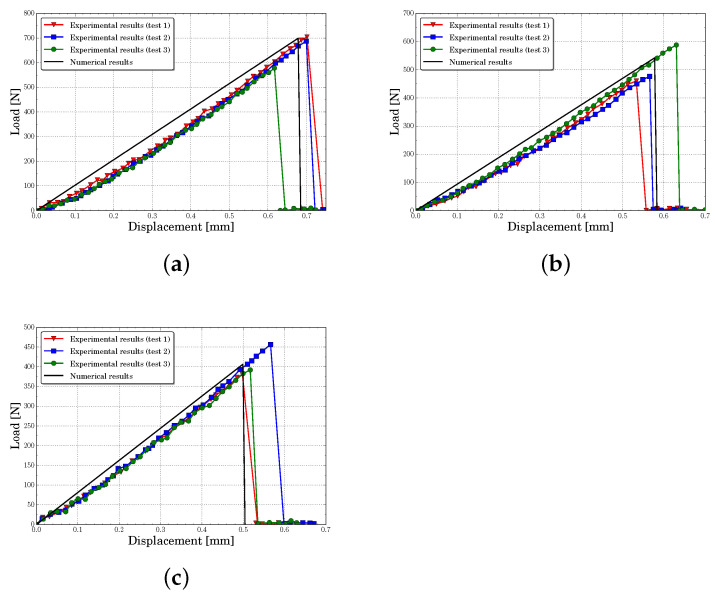
Load-displacement curves for a notch eccentricity of d=20 mm predicted for different notch lengths *a* (**a**) a=6 mm, (**b**) a=8 mm, (**c**) a=10 mm.

**Figure 13:**
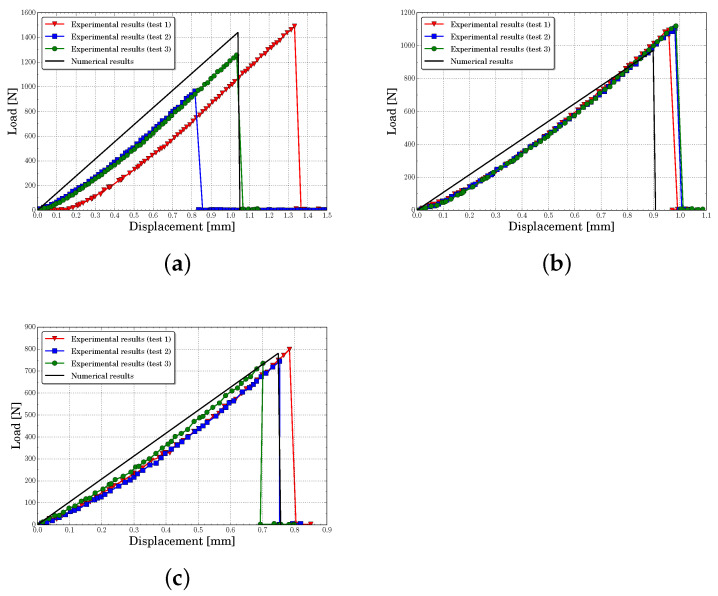
Load-displacement curves for a notch eccentricity of d=30 mm predicted for different notch lengths *a* (**a**) a=6 mm, (**b**) a=8 mm, (**c**) a=10 mm.

Figure 10, Figure 11, Figure 12 and Figure 13 present the experimental and numerical results, in terms of load-displacement curves, for all tested specimens with centered and eccentric notches. We can see that in all cases the curves were described by a bilinear behavior, with a brittle fracture, both numerically and experimentally. Moreover, it can be stated that numerical predictions were in good agreement with the experimental results assuming the experimental dispersion obtained for some specific cases.

Table 3 contains the experimental mean values and the standard deviation of the initial stiffness obtained for each configuration. In this table are included the numerical results and their percent error. The initial stiffness was calculated by means of a least square regression line on pre-peak load-displacement zone curve. It can be observed that the lattice model predicted the initial stiffness with a maximum percent error of 14.2%, being the average error of all cases around 5.6%. The experiments and the numerical results showed that the initial stiffness increased with eccentricity and decreased with notches length.

Table 4 presents the mean peak loads and their standard deviation obtained from the experiments. Furthermore, as in the previous analysis, the experimental data were compared with the numerical results and the percent error was presented. Again, it can be seen that the obtained average percent error considering all analyzed cases was about 7.7%, with a maximum of 17%. Furthermore, the numerical model captured the same tendency of the experiments, where the peak load increased with eccentricity and decreased with notches length.

**Figure 14:**
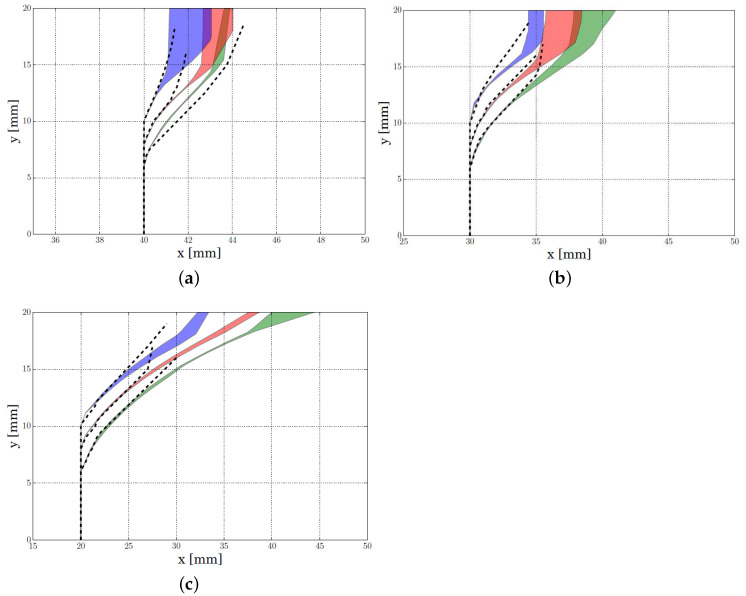
Crack trajectory obtained with the lattice model (in dashed line) and the envelope area of the experimental crack patterns for different initial notch lengths *a*, i.e., green: a=6 mm, red: a=8 mm and blue: a=10 mm and different notch eccentricity *d* (**a**) d=10 mm, (**b**) d=20 mm, (**c**) d=30 mm.

With regard to the crack propagation patterns, Figure 14 compares the patterns obtained with the numerical model and the experimental results, for each notch length and position. The coloured area represents the envelope of the experimental crack patterns obtained for each configuration, while the dash line type corresponds with the numerical results.

The obtained results show that the numerical model was able to capture the crack pattern in the initial fracture process. The difference between the numerical model and experimental results increased when the crack progressed. However, the experimental results showed dispersion increased with the crack advance.

Moreover, in order to present a deeper analysis of the results provided by the proposed model, the initial crack inclination angle $\theta_{0}$ obtained in the crack propagation process for each and every one of the studied geometries was compared with our own experimental results and those obtained by other authors. This initial angle was derived from the values of the stress intensity factor (SIF) in mode I and II.

Munz and Fett [61] defined an analytical expression to calculate these SIFs for the geometry and boundary conditions studied in this work. The geometrical functions of the stress intensity factor can be calculated as:

(18) $$K_{I}=\sigma Y_{I}\sqrt{a},$$

(19) $$K_{II}=\sigma Y_{II}\sqrt{a},$$

being

(20) $$\sigma =\frac{2}{3}\frac{s}{dB^{2}}F,$$

where *a*, *s*, *B* and *d* are the geometrical parameters defined in Figure 3, *F* is the applied load, and $Y_{I}$ and $Y_{II}$ are the normalized geometrical functions extracted from [61]. Table 5 presents the values of the SIF for each test specimen.

**Figure 15:**
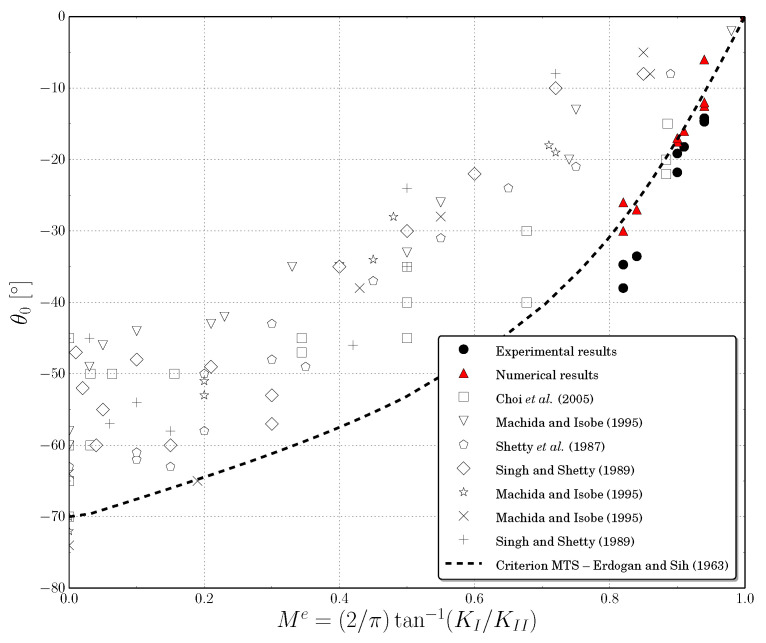
Comparative analysis of the initial crack inclination angle as a function of the mixed fracture mode.

As stated before, Figure 15 compares the initial crack inclination angles $\theta_{0}$ obtained in this work, with own experimental results and those presented by other authors [62,63,64,65].

The angle is presented as a function of the dimensionless parameter $M^{e}$ defined as:

(21) $$M^{e}=\frac{2}{\pi }\tan^{-1}\left(\frac{K_{I}}{K_{II}}\right).$$

Note that this parameter is 0 for a pure mode II, and 1 for a pure mode I. Furthermore, in Figure 15 is presented the analytical curve obtained from the maximum tangential stress (MTS) criterion developed by Erdogan and Sih [66]:

(22) $$\mathrm{sen}\theta_{0}=\frac{K_{II}}{K_{I}}\left(1-3\cos\theta_{0}\right),$$

then

(23) $$\theta_{0}=\tan^{-1}\left[\frac{-3K_{II}\left(\sqrt{8K_{II}^{2}+K_{I}^{2}}+K_{I}\right)}{3K_{II}^{2}+K_{I}\sqrt{8K_{II}^{2}+K_{I}^{2}}}\right],$$

The experimental results showed a maximum dispersion of the inclination angle of about 20°. Note that this dispersion agreed with the results reported by other authors [67]. Moreover, the numerical results obtained with our model had a good correlation with the MTS criterion presented by Erdogan and Sih [66]. The experimental data obtained in this work were lower than the analytical predictions, this difference increased when the $M^{e}$ parameter decreased.

Therefore, in view of the results shown, it can be concluded that the lattice model was able to adequately predict the initial stiffness and peak loads, with average percent errors lower than 8%, and the crack propagation patterns and initial angles on pre-notched PMMA beams subjected to quasi-static three-point bending tests.

## 4. Conclusions and Observations

This paper presents experimental and numerical analysis of the quasi-static fracture behaviour for PMMA, by means of three-point bending tests on specimens with different initial notch lengths and notch eccentricities.

A numerical model consisting in a 2D lattice model based on the Born potential has been developed and validated. The implemented model considers a bilinear constitutive model, with a linear softening law, considering a progressive damage in the material and allowing to overcome the limitation in the selection of the Poisson’s coefficient, present in some other discrete models in the scientific literature.

The validation of the proposed model has been carried out in terms of initial stiffness, peak load, crack propagation patterns and initial crack inclination angle obtained in the conducted three-point-bending tests. The numerical results provide an average error of about 5.6% in the value of initial stiffness, and an average error of about 7.7% in the value of peak load, for all the tested specimens, showing a good agreement with the experimental results. With regard to the crack propagation patterns, the numerical model is able to capture them in the initial fracture process, while the difference between numerical and experimental results increases as the crack progresses, being also the dispersion obtained in the experimental results so much greater at these stages. Finally, with regard to the values of the initial crack propagation angle, the values obtained with the proposed model have a good correlation with the MTS criterion presented by Erdogan and Sih, being the experimental data obtained in this work slightly lower than the analytical predictions.

It can be concluded that in view of the results shown, we have developed a lattice model which allows to select any value of Poisson’s coefficient and is able to predict the initial stiffness, peak load, crack propagation pattern and initial crack inclination angle of PMMA specimens with different initial notch lengths and notch eccentricities subjected to three-point bending tests under quasi-static load conditions.

Future works are required to include a parametric study with the proposed model. In this way, we can study an upper range of the dimensionless parameter $M^{e}$ and their effect on the crack inclination angle. Moreover, it could be interesting to include the plasticity in the constitutive model of the proposed discrete LM, incorporating also strain rate dependency. Furthermore, the random nature of the material can be studied by using an aleatory distribution of mechanical properties associated with each bond. Additional work is also required to extend the proposed model to a general three-dimensional case or to validate the capability of the lattice model to predict the crack propagation velocities.

## Figures and Tables

**Table 1:** Fracture toughness test results.

Test	$P_{Q}$	$K_{\mathrm{IC}}$
01	214.3 N	1.61 MPa$\sqrt{m}$
02	229.2 N	1.73 MPa$\sqrt{m}$
03	206.3 N	1.51 MPa$\sqrt{m}$

**Table 2:** Mechanical properties of polymethyl methacrylate (PMMA).

Properties	Values
Density [23]	1190.0 Kg/$m^{3}$
Young’s modulus	2842 MPa
Poisson ratio [23]	0.401
Tensile strength	56.8 MPa
Fracture energy	775.37 N/m

**Table 3:** Comparison of the initial stiffness obtained from experimental and numerical analysis.

*d* [mm]	*a* [mm]	Experimental [N/mm]	Numerical [N/mm]	Error [%]
0	6	765.9 ± 14.7	821.5	7.3
	8	695.0 ± 18.2	661.7	4.8
	10	502.3 ± 34.4	485.3	3.4
10	6	818.1 ± 35.8	913.4	11.6
	8	727.6 ± 63.6	746.5	2.6
	10	565.8 ± 82.6	597.9	5.7
20	6	990.6 ± 43.1	1030.9	4.1
	8	899.1 ± 37.4	938.4	4.4
	10	798.0 ± 26.7	814.0	2.0
30	6	1214.0 ± 19.1	1386.0	14.2
	8	1146.4 ± 11.4	1081.0	5.7
	10	1026.0 ± 23.8	1043.3	1.7

**Table 4:** Comparison of the peak loads obtained from experimental and numerical analysis.

*d* [mm]	*a* [mm]	Experimental $P_{Q}$ [N]	Numerical $P_{Q}$ [N]	Error [%]
0	6	370.5 ± 17.6	345.8	6.7
	8	353.4 ± 26.3	292.8	17.1
	10	219.5 ± 9.0	216.6	1.3
10	6	411.3 ± 19.0	458.6	11.5
	8	387.2 ± 46.9	356.6	7.9
	10	247.6 ± 61.3	266.0	7.4
20	6	674.5 ± 73.6	699.0	3.6
	8	522.8 ± 59.7	542.0	3.7
	10	393.1 ± 50.9	406.5	3.4
30	6	1359.9 ± 111.5	1440.4	12.4
	8	1125.7 ± 55.7	970.8	12.9
	10	743.7 ± 40.3	781.1	5

**Table 5:** Stress intensity factor in mode I and II.

*d* [mm]	*a* [mm]	$Y_{I}$	$Y_{\mathrm{II}}$	$K_{I}$ [MPa$\sqrt{m}$]	$K_{\mathrm{II}}$ [MPa$\sqrt{m}$]
0	6	0.0058	-	2.195 ± 0.102	-
	8	0.0067	-	2.252 ± 0.175	-
	10	0.0074	-	1.634 ± 0.067	-
10	6	1.5114	0.1391	1.445 ± 0.032	0.133 ± 0.003
	8	1.6895	0.1703	1.114 ± 0.159	0.131 ± 0.019
	10	2.0086	0.1988	1.825 ± 0.461	0.108 ± 0.030
20	6	1.0062	0.1414	1.397 ± 0.153	0.252 ± 0.028
	8	1.1235	0.1740	1.576 ± 0.180	0.244 ± 0.028
	10	1.3343	0.2019	1.911 ± 0.226	0.247 ± 0.029
30	6	0.5172	0.1314	1.282 ± 0.105	0.530 ± 0.043
	8	0.5690	0.1688	1.544 ± 0.022	0.548 ± 0.008
	10	0.6719	0.1986	1.545 ± 0.081	0.457 ± 0.024

## Data Availability

The data presented in this study are available on request from the corresponding author.
